# Dynamic changes of functional fitness, antibodies to SARS-CoV-2 and immunological indicators within 1 year after discharge in Chinese health care workers with severe COVID-19: a cohort study

**DOI:** 10.1186/s12916-021-02042-0

**Published:** 2021-07-14

**Authors:** Lijuan Xiong, Qian Li, Xiongjing Cao, Huangguo Xiong, Ming Huang, Fengwen Yang, Qingquan Liu, Daquan Meng, Mei Zhou, Gang Wang, Jun Tong, Tengfei Chen, Yanzhao Zhang, Xinliang He, Yunzhou Fan, Yupeng Zhang, Liang Tang, Yang Jin, Jiahong Xia, Yu Hu

**Affiliations:** 1 Department of Nosocomial Infection Management, Union Hospital, Tongji Medical College, Huazhong University of Science and Technology, Wuhan, Hubei China; 2 Institute of Hematology, Union Hospital, Tongji Medical College, Huazhong University of Science and Technology, Wuhan, 430022 Hubei China; 3 State Key Laboratory of Component-Based Chinese Medicine, Tianjin University of Traditional Chinese Medicine, Tianjin, China; 4 Evidence-Based Medicine Center, Tianjin University of Traditional Chinese Medicine, Tianjin, China; 5 CCM, Medicine Bachelor Beijing Hospital of Traditional Chinese Medicine, Capital Medical University, Beijing, China; 6 Department of Respiratory and Critical Care Medicine, NHC Key Laboratory of Pulmonary Diseases, Union Hospital, Tongji Medical College, Huazhong University of Science and Technology, Wuhan, 430022 Hubei China; 7 Department of Rehabilitation, Union Hospital, Tongji Medical College, Huazhong University of Science and Technology, Wuhan, Hubei China; 8 Affiliated Wuhan Mental Health Center, Tongji Medical College, Huazhong University of Science and Technology, Wuhan, Hubei China; 9 Department of Cardiovascular Surgery, Union Hospital, Tongji Medical College, Huazhong University of Science and Technology, Wuhan, 430022 Hubei China

**Keywords:** Novel coronavirus, COVID-19, Antibody, Cytokine, Lymphocyte subsets

## Abstract

**Background:**

Few studies had described the health consequences of patients with coronavirus disease 2019 (COVID-19) especially in those with severe infections after discharge from hospital. Moreover, no research had reported the health consequences in health care workers (HCWs) with COVID-19 after discharge. We aimed to investigate the health consequences in HCWs with severe COVID-19 after discharge from hospital in Hubei Province, China.

**Methods:**

We conducted an ambidirectional cohort study in “Rehabilitation Care Project for Medical Staff Infected with COVID-19” in China. The participants were asked to complete three physical examinations (including the tests of functional fitness, antibodies to SARS-CoV-2 and immunological indicators) at 153.4 (143.3, 164.8), 244.3 (232.4, 259.1), and 329.4 (319.4, 339.3) days after discharge, respectively. Mann-Whitney U test, Kruskal-Wallis test, t test, one-way ANOVA, χ^2^, and Fisher’s exact test were used to assess the variance between two or more groups where appropriate.

**Results:**

Of 333 HCWs with severe COVID-19, the HCWs’ median age was 36.0 (31.0, 43.0) years, 257 (77%) were female, and 191 (57%) were nurses. Our research found that 70.4% (114/162), 48.9% (67/137), and 29.6% (37/125) of the HCWs with severe COVID-19 were considered to have not recovered their functional fitness in the first, second, and third functional fitness tests, respectively. The HCWs showed improvement in muscle strength, flexibility, and agility/dynamic balance after discharge in follow-up visits. The seropositivity of IgM (17.0% vs. 6.6%) and median titres of IgM (3.0 vs. 1.4) and IgG (60.3 vs. 45.3) in the third physical examination was higher than that in the first physical examination. In the third physical examination, there still were 42.1% and 45.9% of the HCWs had elevated levels of IL-6 and TNF-α, and 11.9% and 6.3% of the HCWs had decreased relative numbers of CD3^+^ T cells and CD4^+^ T cells.

**Conclusion:**

The HCWs with severe COVID-19 showed improvement in functional fitness within 1 year after discharge, active intervention should be applied to help their recovery if necessary. It is of vital significance to continue monitoring the functional fitness, antibodies to SARS-CoV-2 and immunological indicators after 1 year of discharge from hospital in HCWs with severe COVID-19.

**Supplementary Information:**

The online version contains supplementary material available at 10.1186/s12916-021-02042-0.

## Background

Since December 2019, coronavirus disease 2019 (COVID-19) caused by severe acute respiratory syndrome coronavirus 2 (SARS-CoV-2) break out in Wuhan City, Hubei Province, China. Subsequently, many people in other countries worldwide were found to be infected with the respiratory infectious disease. As of June 15, 2021, COVID-19 had caused over 175 million confirmed cases and more than 3.8 million deaths, posing an important threat to the lives and health of the global population [1]. Health care workers (HCWs) faced a relatively higher risk of SARS-CoV-2 infection in the fight against COVID-19. According to the data as of February 11, 2020, the number of HCWs with COVID-19 was 3019 (1716 confirmed cases) [2]. Among the confirmed cases in HCWs, around two thirds (64%, 1088/1688) of them are from Hubei Province where SARS-CoV-2 was first detected [2]. Additionally, the proportion of HCWs with severe COVID-19 in Hubei Province especially Wuhan is the highest in China. The health consequences of these HCWs with severe COVID-19 in Hubei Province after discharge from hospital have attracted worldwide attention and need to be evaluated urgently.

So far, studies focusing on dynamic changes of functional fitness, antibodies to SARS-CoV-2, and immunological indicators in patients with COVID-19 after discharge from hospital are scarce. Previous studies had investigated functional fitness [3], antibodies to SARS-CoV-2 [4], and immunological indicators [5–9] in patients with COVID-19 after SARS-CoV-2 infection. Baricich et al. reported that 32% (66/204) of the Italian patients with COVID-19 had an impaired functional fitness performance at 3~6 months after discharge from hospital [3]. In that study, the functional fitness was evaluated using the Short Physical Performance Battery test. Other tests utilized to assess the recovery of functional fitness included 6-min walking test and the Senior Fitness Test (SFT). The SFT was first developed for the elderly [10]; however, a recent study revealed its potential use in various age group [11]. The recovery of functional fitness using SFT in patients with COVID-19 remains unclear. Dan et al. [12] and Hartley et al. [13] found that patients retained immune memory after SARS-CoV-2 infection between 4 to 242 days post-symptom onset and at approximately 6 months after infection, respectively. The dynamic changes of immunological indicators after SARS-CoV-2 infection or discharge were rarely known. The sample sizes of these studies ranged from 25 to 1733, and the follow-up time ranged from 4 days after onset of symptoms to 8 months after SARS-CoV-2 infection. To the authors’ knowledge, no research has yet focused on dynamic changes of functional fitness, antibodies to SARS-CoV-2, and immunological indicators after discharge from hospital in the population of HCWs, and studies focusing on longer follow-up time are warranted. The proportion of HCWs with severe COVID-19 is the highest in Hubei Province in China. The health consequences of these HCWs with severe COVID-19 after discharge are the focus of global attention.

Therefore, for the first time, this research aimed to report the dynamic changes of functional fitness, antibodies to SARS-CoV-2 and immunological indicators in HCWs with severe COVID-19 after discharge from hospital within 1 year in Hubei Province, China.

## Methods

### Study design and participants

The participants in this cohort study came from “Rehabilitation Care Project for Medical Staff Infected with COVID-19” in China. The project was initiated by the Chinese Academy of Engineering and Tencent Charity Foundation, aiming to investigate the health consequences (including mental and physical) in HCWs with COVID-19 after discharge from hospital.

So far, the project has carried out follow-up studies up to 1 year after discharge covering psychological evaluation and intervention, survey of persistent symptoms, lung function evaluation and rehabilitation intervention, physical rehabilitation evaluation and intervention, physical examinations, consultation with academicians, and expert teams and online lectures.

In the participants of “Rehabilitation Care Project for Medical Staff Infected with COVID-19” in China, in this study, participants with the following criteria were excluded: (1) without admission information, (2) missing information on comorbidities, and (3) mild or moderate COVID-19. The disease severity in HCWs was assessed according to the guidelines recommended by the National Health Commission [14]. The HCWs were not allowed to discharge unless meeting the all standards recommended by the National Health Commission [14].

This study was approved by the Ethics Committee of Union Hospital, Tongji Medical College, Huazhong University of Science and Technology (2020-0506) according to the principles of the Declaration of Helsinki. Each participant in this study provided informed written consent to participate with this project.

### Procedures

Information on demographic (such as age, sex, education, roles in work, work experience, location of the hospital work for, body-height, body weight, smoke habit) and clinical characteristic (such as symptoms at admission, ICU admission, comorbidities) of the HCWs was obtained at enrollment.

The HCWs with severe COVID-19 were asked to participate in the first (from July to August 2020), second (from October to November 2020), and third physical examinations (January 2021) in the Physical Examination Center of Union Hospital (Tongji Medical College, Huazhong University of Science and Technology). As of January 2021, 218, 217, and 209 HCWs had completed the first, second, and third physical examination, respectively, and the longest follow-up time can be up to 1 year since discharge from hospital. In each physical examination, functional fitness tests were performed, and blood samples of the HCWs were collected.

Functional fitness test was evaluated using the SFT which included assessment of muscle strength (30-s arm curl test and 30-s chair stand), flexibility (back scratch test, modified trunk rotation and chair sit-and-reach test), and agility/dynamic balance (stance with eyes and functional reach test) [10]. The reasons for choosing the existing functional fitness test are listed below: (1) the SFT test could comprehensively reflect the physical recovery of the participants with respect to muscle strength, flexibility, and agility/dynamic balance; (2) acceptable reliability (ICC > 0.7) was observed for SFT and other functional fitness test including 6-min walking test and SFT could also applied to other population beyond the elderly [11]; and (3) STF needs less time to perform and was found to be convenient for both the participants and the investigators [15]. The functional fitness test was conducted under the professional guidance of doctors in Physical Medicine and Rehabilitation Department of Union Hospital (Tongji Medical College, Huazhong University of Science and Technology). If the HCWs could not or obviously had difficulty in completing the test, the HCWs were considered to have not recovered their functional fitness by the doctors.

The blood samples were used for the determination of antibodies to SARS-CoV-2 and immunological indicators in the Department of Laboratory Medicine of Union Hospital (Tongji Medical College, Huazhong University of Science and Technology). In this study, N protein was coated on the plate for the serum IgM (100 μl, dilution factor was 1:100) and IgG (100 μl, dilution factor was 1:20) enzyme-linked immunosorbent assay. The ELISA kits of Livzon Diagnostics Inc., Zhuhai, China, were used to evaluate the serum immunoglobulin IgM/IgG antibodies against SARS-CoV-2. The methods of detection were described in detail in previous study [16]. If the titres of antibodies in HCWs were greater than 10, HCWs were considered to have positive antibodies against SARS-CoV-2. We assigned the value 5 to 21 and 16 HCWs with seronegativity of antibodies but no exact antibody titre in the first and third physical examination, respectively. In the second physical examination, some HCWs only tested the seropositivity of antibodies and did not perform the titre tests due to the lack of quantitative detection kits. Therefore, only the antibody titres between the first and third physical examinations were compared in this study.

In the present study, in addition to comparing the levels of cytokines and lymphocyte subsets in HCWs during the three physical examinations, 30 HCWs were also enrolled who were admitted to Union Hospital (Tongji Medical College, Huazhong University of Science and Technology) due to SARS-CoV-2 infection (30 and 28 had levels of cytokines and lymphocyte subsets before discharge, respectively). The immunological indicators of these HCWs were measured in the same laboratory (Department of Laboratory Medicine, Union Hospital) using the same method. The BD™ Cytometric Bead Array (CBA) Human Th1/Th2 cytokine kit was used in the measurement of Th1/Th2 inflammatory cytokines. The levels of cytokine profile (IFN-γ, IL-10, IL-2, IL-4, IL-6, and TNF-α) were quantified by BD cytometric bead array analysis. Flow cytometry was performed using a BD FACSCanto™ (BD Biosciences) in lymphocyte subsets (B cells, CD3^+^ T cells, CD4^+^ T cells, CD4^+^/CD8^+^ cell ratio, CD8^+^ T cells, and NK cells) detection, and data were analysed with FCAP version 3.0 software.

### Statistical analysis

In this study, median (IQR) was used to describe the continuous covariate (including age, days after discharge from hospital, and measurements of the blood samples). Number (%) was utilized to show the categorical covariate. The variance of covariates in two or more than two groups was evaluated using Mann-Whitney U test, Kruskal-Wallis test, one-way ANOVA, t test, χ^2^, and Fisher’s exact test where appropriate. Pearson correlations were calculated between levels of cytokines and levels of lymphocyte subsets. All the analyses in this study were conducted with R version 4.0.3 and SAS version 9.4 (SAS Institute, Cary, NC). A two-sided *P* value < 0.05 was considered statically significant in this study.

## Results

**Fig. 1:**
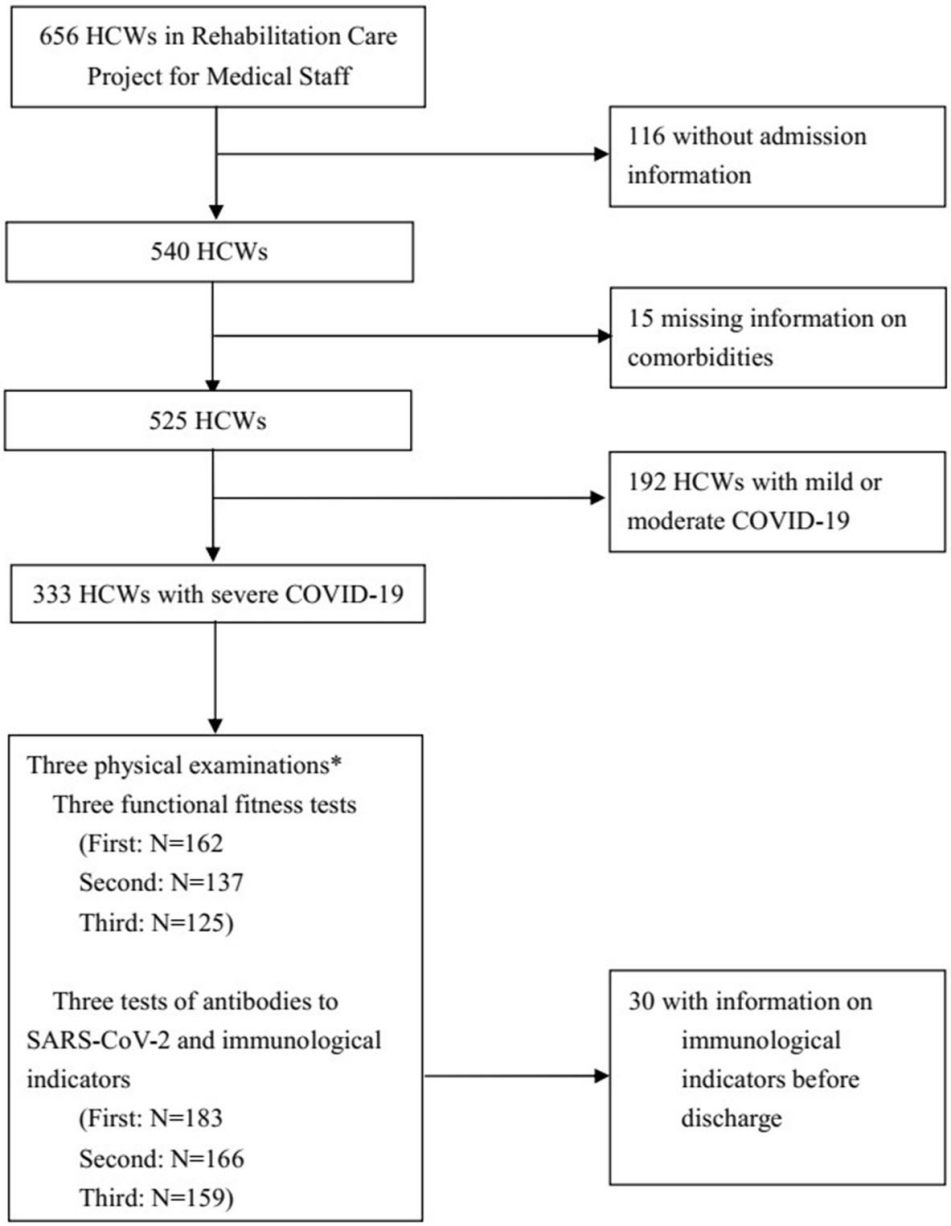
Flow chart of HCWs with COVID-19 in Rehabilitation Care Project for Medical Staff Infected with COVID-19 in China. COVID-19, novel coronavirus pneumonia; HCWs, health care workers. Asterisk indicates the three physical examinations (including tests of functional fitness, antibodies to SARS-CoV-2, and immunological indicators) were conducted at 153.4 (143.3, 164.8), 244.3 (232.4, 259.1), and 329.4 (319.4, 339.3) days after discharge

A total of 656 HCWs with COVID-19 were enrolled in the cohort. They discharged from the hospitals from January 22, 2020, to May 25, 2020. In this study, 116 HCWs without admission information and 15 HCWs without information on comorbidities were excluded. Of the remaining 525 HCWs who discharged from hospital since January 22, 2020, to May 25, 2020 (Additional file 1: Fig. S1), after exclusion of 192 HCWs with mild or moderate COVID-19, 333 HCWs with severe COVID-19 were enrolled in this study. At 153.4 (143.3, 164.8), 244.3 (232.4, 259.1), and 329.4 (319.4, 339.3) days after discharge, the HCWs were asked to complete three physical examinations. A total of 162, 137, and 125 HCWs performed the first, second, and third functional fitness tests; 183, 166 and 159 HCWs underwent the first, second, and third test of immunological indicators, respectively. A sum of 30 HCWs had information on immunological indicators before discharge (from symptom onset to discharge), and these 30 HCWs were involved in the analyses of dynamic changes of immunological indicators before discharge and in three physical examinations (Fig. 1).

The demographic and clinical characteristics of 333 HCWs with severe COVID-19 were presented in Table 1. In this cohort, the HCWs’ median age was 36.0 (31.0, 43.0) years; the majority of the HCWs were female (257, 77%), were nurses (191, 57%), had college or higher degree (252/327, 77%), and had work experience of more than 10 years (206, 62%). The top two clinical symptoms in HCWs at admission were fatigue (243, 73%) and fever (239, 72%). About 5% (17/328) of the HCWs had been in intensive care unit (ICU) and nearly one third (103, 31%) of them had comorbidities. The demographic characteristics of HCWs included and excluded in this study were presented in Additional file 1: Table S1.

**Table 1:** Demographic and clinical characteristics of HCWs with severe COVID-19

Characteristics	All (N = 333)
**Demographic characteristics**	
Age (years)	36.0 (31.0–43.0)
Sex	
Male	76 (23%)
Female	257 (77%)
Education	
High school and lower	75/327 (23%)
College and higher	252/327 (77%)
Work experience(years)	
< 5	38 (11%)
5–10	89 (27%)
> 10	206 (62%)
Location of the hospital work for	
Hankou, Wuhan	184 (55%)
Wuchang, Wuhan	80 (24%)
Hanyang, Wuhan	22 (7%)
Outside Wuhan in Hubei	47 (14%)
Roles in work	
Doctors	89 (27%)
Nurses	191 (57%)
Other	53 (16%)
BMI (kg/m^2^)	23.5 (21.1–25.9)
Smoke habit	
No	326 (98%)
Yes	7 (2%)
**Clinical characteristics**	
Severity of COVID-19	
Mild or moderate	0 (0%)
Severe	333 (100%)
Symptoms at admission	
Fatigue	243 (73%)
Fever	239 (72%)
Muscle soreness	153 (46%)
Cough	136 (41%)
Chest distress	133 (40%)
Dry cough	122 (37%)
Shortness of breath	117 (35%)
Diarrhoea	98 (29%)
Headache	84 (25%)
Dyspnoea	73 (22%)
Vomiting	31 (9%)
ICU admission	
No	311/328 (95%)
Yes	17/328 (5%)
Comorbidities	
No	230 (69%)
Yes	103 (31%)

Data are n (%), n/N (%), or median (IQR). The differing denominators used indicate missing data. *BMI* body mass index, *COVID-19* novel coronavirus pneumonia, *ICU* intensive care unit admission

A total of 162, 137, and 125 HCWs underwent functional fitness tests during the three physical examinations. In this study, 70.4% (114/162), 48.9% (67/137), and 29.6% (37/125) of the HCWs with severe COVID-19 were considered to have not recovered their functional fitness in the first, second, and third functional fitness tests, respectively. Comparing the scores in the three functional fitness tests, this present research found that the variance of scores in the muscle strength test (30-s arm curl test and 30-s chair stand), flexibility test (modified trunk rotation), and agility/dynamic balance (left leg stance with eyes open, right leg stance with eyes open, left leg stance with eyes closed, right leg stance with eyes closed, and functional reach test) in three physical examinations was statistically significant (Table 2).

**Table 2:** Results of functional fitness in three physical examinations

Categories	Physical examination			***P***
	First (N = 162)	Second (N = 137)	Third (N = 125)	
**Muscle strength test**				
30-s arm curl test, n	17.0 (14.0, 20.0)	20.0 (17.0, 23.0)	23.0 (19.0, 26.0)	< 0.001*
30-s chair stand, n	15.0 (13.0, 17.0)	17.0 (15.0, 19.0)	17.0 (15.0, 20.0)	< 0.001*
**Flexibility test**				
Back scratch test (left)	0.3 (− 8.0, 3.0)	0.00 (− 8.4, 3.2)	− 1.1 (− 8.0, 2.3)	0.59
Back scratch test (right)	2.3 (− 2.4, 5.0)	1.7 (− 0.8, 6.0)	2.1 (1.7, 4.7)	0.85
Modified trunk rotation (°)	34.7 (28.5, 39.6)	44.2 (36.5, 52.0)	42.2 (34.6, 47.4)	< 0.001*
Chair sit-and-reach test, cm	1.2 (− 4.0, 7.6)	3.0 (− 2.6, 9.3)	2.1 (− 3.8, 7.8)	0.53
**Agility/dynamic balance**				
Left leg stance with eyes open	60.0 (60.0, 60.0)	60.0 (60.0, 60.0)	60.0 (60.0, 60.0)	< 0.001*
Right leg stance with eyes open	60.0 (60.0, 60.0)	60.0 (60.0, 60.0)	60.0 (60.0, 60.0)	< 0.001*
Left leg stance with eyes closed	6.1 (1.0, 16.0)	11.0 (5.5, 24.2)	13.6 (6.0, 28.7)	< 0.001*
Right leg stance with eyes closed	8.6 (3.0, 18.1)	14.0 (6.0, 26.0)	13.0 (6.0, 20.0)	< 0.001*
Functional reach test, cm	27.8 (6.8, 31.2)	27.7 (24.0, 32.5)	29.6 (24.6, 32.3)	< 0.001*

Data are median (IQR). The comparison of scores in functional fitness test in the first, second, and third physical examination was performed with Kruskal-Wallis test. *P <0.05

In this study, the seropositivity of IgM were 6.6% (12/183), 10.8% (18/166), and 17.0% (27/159) in the first, second, and third physical examination, respectively. The seropositivity of IgG were 89.6% (164/183), 86.7% (144/166), and 91.2% (145/159) in the first, second, and third physical examination, respectively. Compared with the seropositivity of IgM in the first physical examination, the seropositivity of IgM in the third physical examination was higher (*P* value = 0.003). There was no statistically significant difference in the seropositivity of IgG in the first, second, and third physical examinations.

**Fig. 2:**
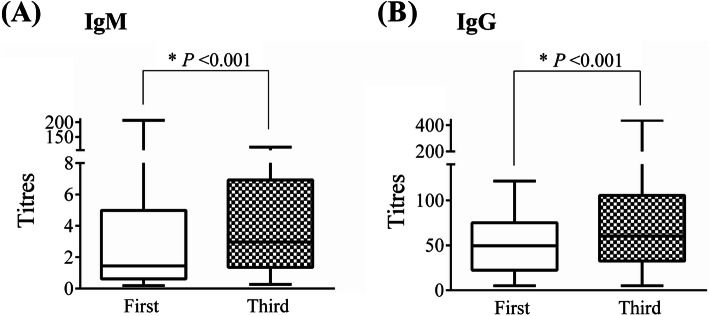
Antibody titres against SARS-CoV-2 in the first and third physical examination. **A** The titres of IgM in the first and third physical examination. **B** The titres of IgG in the first and third physical examination. The comparison of titres of IgM and IgG in the first and third physical examination was performed with Mann-Whitney U test. The sample sizes in the first and third physical examination were 183 and 159, respectively. Data are shown as min, Q25, Q50, Q75, and max value. *P < 0.001

In this study, the HCWs who did the second physical examinations on date of October 15, 2020, and October 17, 2020, only had the results of seropositivity of the IgM/IgG antibodies, missing the titres of antibodies. Therefore, only the titres of antibodies in the first and third physical examination were compared. It was found that the median titres of antibodies in the third physical examination were significantly higher than that in the first physical examination (IgM 3.0 vs. 1.4, *P* value < 0.001; IgG 60.3 vs. 45.3, *P* value < 0.001) (Fig. 2). To verify the reliability of the results, the analyses was repeated in 136 HCWs who had titres of antibodies both in the first and the third physical examinations, and found the results were robust (*P* value < 0.001) (Additional file 1: Fig. S2).

**Fig. 3:**
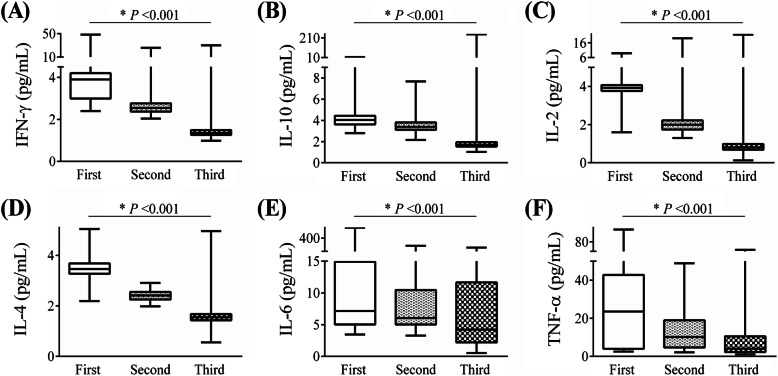
Levels of cytokine profile categorized by the time of physical examination. A IFN-γ, **B** IL-10, **C** IL-2, **D** IL-4, **E** IL-6, and **F** TNF-α. The median levels of cytokines in the first, second, and third physical examination were compared using Kruskal-Wallis test. The sample sizes in the first, second, and third physical examination were 183, 166, and 159, respectively. Data are shown as min, Q25, Q50, Q75, and max value. *P < 0.001

Comparing the median values of cytokines in three physical examinations, the study found that the differences in median values of all six cytokine indicators (IFN-γ, IL-10, IL-2, IL-4, IL-6, and TNF-α) were statistically significant (Fig. 3, all *P* value < 0.001). With the extension of the time of discharge, the cytokine levels in the HCWs showed a downward trend. The analyses were repeated among 114 HCWs with all three cytokine measurements and it was also found that a significant decline trend of all six cytokine indicators (Additional file 1: Fig. S3, all *P* value < 0.001). As presented in Additional file 1: Table S2, current results showed that cytokines including IL-6, IL-4, TNF-α, and IL-2 measured in the first physical examination were significantly affected. In the third physical examination, it was mainly manifested as an increase in the levels of IL-6 (42.1%) and TNF-α (45.9%) in the HCWs. The distribution and levels of cytokine before discharge and in the three physical examinations in 30 HCWs were presented in Additional file 1: Table S3 and S4. Compared with the levels of cytokines before discharge, the levels of IFN-γ, IL-2, and IL-4 first increased and then decreased after discharge, reaching the peaks at the first physical examination. The levels of IL-10 and IL-6 decreased after discharge. The levels of IL-10 decreased since the second physical examination, and the levels of IL-6 decreased at the third physical examination (Additional file 1: Table S4).

**Fig. 4:**
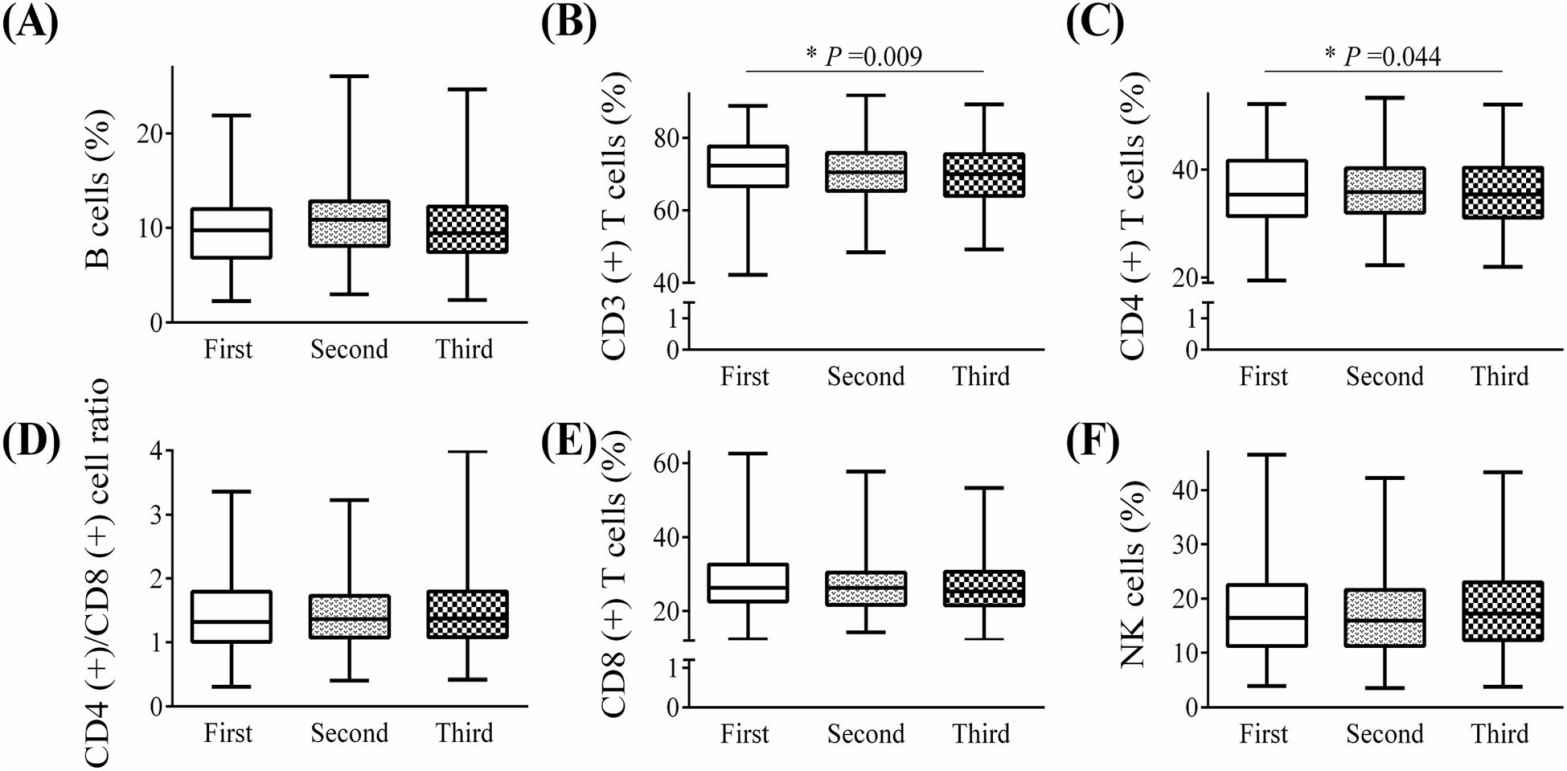
Relative numbers of lymphocyte subsets categorized by the time of physical examination. **A** B cells, **B** CD3^+^ T cells, **C** CD4^+^ T cells, **D** CD4^+^/CD8^+^ cell ratio, **E** CD8^+^ T cells, and **F** NK cells. The median relative numbers of lymphocyte subsets in the first, second, and third physical examination were compared using Kruskal-Wallis test. The sample sizes in the first, second, and third physical examination were 183, 166, and 159, respectively. Data are shown as min, Q25, Q50, Q75, and max value. *P < 0.05

Comparing the median values of lymphocyte subsets in three physical examinations in HCWs, this study found significant variance of indications including CD3^+^ T cells (*P* value = 0.009) and CD4^+^ T cells (*P* value = 0.044) in three physical examinations, but not B cells, CD4^+^/CD8^+^ cell ratio, CD8^+^ T cells, and NK cells (Fig. 4). Analyses were repeated in 114 HCWs who had all three lymphocyte subsets measurements; no significant difference was observed (Additional file 1: Fig. S4, all *P* value > 0.05). According to Additional file 1: Table S5, the most affected indicators included CD8^+^ T cells (9.8% elevated), NK cells (9.3% elevated), and CD3^+^ T cells (8.7% decreased) in the first physical examination. In the third physical examination, the abnormalities in indicators mainly included NK cells (11.3% elevated), CD3^+^ T cells (11.9% decreased), and CD4^+^ T cells (6.3% decreased). The distribution and relative numbers of lymphocyte subsets before discharge and in the three physical examinations in 28 HCWs were shown in Additional file 1: Table S6 and S7. The results of this study showed that the relative numbers of CD4^+^ T cells showed a trend of declining while relative numbers of NK cells showed a trend of increasing over time after discharge.

As presented in Additional file 1: Table S8, results revealed non-significant associations between indicators of cytokines and lymphocyte subsets (all r values ranged from − 0.086 to 0.097, all *P* value > 0.05).

## Discussion

This is the first study on the dynamic changes of functional fitness, antibodies to SARS-CoV-2, and immunological indicators within 1 year after discharge in HCWs with severe COVID-19. In this study, 70.4%, 48.9%, and 29.6% of the HCWs with severe COVID-19 were reported that their functional fitness had not recovered in the first, second, and third functional fitness test. In the three physical examinations, the HCWs with severe COVID-19 showed improvement in muscle strength, flexibility and agility/dynamic balance. Compared with the results in the first physical examination, the seropositivity of IgM and titres of IgM/IgG in the third physical examination were higher in HCWs with severe COVID-19. In the third physical examination, there still were 42.1% and 45.9% of the HCWs had elevated levels of IL-6 and TNF-α, and 11.9% and 6.3% of the HCWs had decreased relative numbers of CD3^+^ T cells and CD4^+^ T cells. This study showed that HCWs with severe COVID-19 exhibited improvement in functional fitness within 1 year after discharge. Nearly half of the HCWs with severe COVID-19 still had abnormal immunological indicators. The results suggest the need to continue monitoring the functional fitness, antibodies to SARS-CoV-2, and immunological indicators after 1 year of discharge from hospital in HCWs with severe COVID-19.

This study found 70.4% of the HCWs with severe COVID-19 had not recovered their functional fitness in the first functional fitness tests. The proportion of 70.4% is higher than the proportion (32%) reported in a cross-sectional study in patients with COVID-19 at 3~6 months after discharge [3]. Compared with that study, the study population in present study are younger (median age of present study, 36.0 years; mean age of that study, 57.9 years) and all the participants are severely ill in the current study [3]. In addition to the decline in functional fitness found in patients with COVID-19, patients infected with SARS had also been found to have decreased functional fitness, which may persist up to 1–2 years after infection [17]. It was found that as the discharge time increased, the scores of functional fitness in HCWs showed a trend of improvement, but active rehabilitation interventions including exercise [18] were also urgently needed to help their physical recovery [19]. The specific mechanisms of the decline in functional fitness due to infection are still unclear. Some studies suggested that the decline in functional fitness after infection may be related to prolonged periods of immobility [20] or impaired neuromuscular function [21], but more follow-up studies are still warranted.

The seropositivity of IgG was found to be about 90% in all the three physical examinations, which was similar to the positive rate of IgG (> 90% for N-IgG, RBD-IgG, and S-IgG) reported in Chinese patients with COVID-19 at six months after discharge [4]. The current results showed that compared with the results in the first physical examination, the seropositivity of IgM and titres of IgM/IgG in the third physical examination was higher. Sun et al. also found elevated levels of IgM and IgG antibodies as the time after onset of symptoms increased in COVID-19 patients who were not admitted to the ICU [22]. Levels of IgM reached the highest at 2 weeks after the onset of symptoms, and levels of IgG showed an increasing trend 3 weeks after the onset of symptoms [22]. Jin et al. found that the seropositivity of IgM first increased slightly and then showed a downward trend, and the seropositivity of IgG antibody showed an upward trend and then gradually stabilized since symptom onset to 55 days later [23]. The increase in the seropositivity and titres of IgM antibody in this study raises concern about SARS-CoV-2 re-infection, the prevention and surveillance of SARS-CoV-2 re-infection in the population should be strengthened.

Before this research, previous studies had also found cytokine storm in patients infected with SARS-CoV [24], H5N1 [25], and H7N9 [26]. Consistent with present study results, Wan et al. found elevated IL-6 and IL-10 in Chinese patients with severe COVID-19 compared with patients with mild COVID-19 [8]. Qin et al. reported higher IL-2, IL-6, IL-8, IL-10, and TNF-α in Chinese patients of severe COVID-19 group than patients of mild group [6]. Elevated levels of these cytokines were considered to be manifestations of cytokine storms (a kind of uncontrolled systemic inflammatory reaction process, the characteristic included promotion of excessive release of inflammatory cytokines, causing severe immune pathological damage). In the current study, the levels of cytokines including IL-6 and IL-10 in HCWs showed a trend of decrease over time after discharge from hospital. This suggested that the HCWs’ cytokine indicators showed signs of improvement. However, according to the results in this study, there still were nearly half of the HCWs had elevated cytokines nearly 1 year after discharge. This suggested that the cytokine levels should continue to be closely monitored in HCWs with severe COVID-19 after 1 year discharge from hospital. More studies are warranted to investigate the mechanism of abnormal cytokine levels in HCWs with severe COVID-19 in the future.

In this study, the abnormal lymphocyte subsets was mainly manifested as a decrease in NK cells, CD3^+^ T cells, and CD4^+^ T cells before discharge which had been found in patients infected with SARS-CoV-2 [7, 9] and SARS-CoV [27]. Similar to the present study, Chinese patients with COVID-19 of severe group were found to have decreased lymphocyte subsets comparing with mild group [6, 8]. In patients infected with SARS-CoV, decreases in CD4^+^ T cells, CD8^+^ T cells, B cells, and NK cells were observed in 100%, 87%, 76%, and 55% of the study population at acute stage, respectively [27]. Interestingly, the abnormality of NK cells before discharge was mainly manifested as a decrease in NK cells, but in the follow-up after discharge, the abnormality of NK cells was mainly manifested as an increase instead, which has rarely been reported in other literature. The decrease of lymphocyte subsets was considered to be the main manifestation of viral infection; we speculate that the observed decrease in CD3^+^ T cells in this study might be related to the decrease of CD4^+^ T cells and the increase of CD8^+^ T cells. Some studies observed that lymphocyte subsets were negatively correlated with the levels of IL-6 and IL-8, and IL-6 levels were usually elevated in patients after SARS-CoV-2 infection, especially in severe patients [5]. In the present study, significant correlations were not found between cytokines and lymphocyte subsets. Some studies hypothesized that lymphocytopenia might be caused by cytokine-induced T cell depletion or might be associated with lymphocyte infiltration mobilized by cytokine storm [5]. The specific mechanism remains unclear.

This research has several limitations that deserve attention. Firstly, SFT was used as the assessment of functional fitness test. The method may also be applicable in middle-aged population though the method was originally designed for the elderly [11]. Subsequent research including other tests such as the 6-min walking test, Barthel Index, functional independence measure, modified Rankin scale, timed up and go test, or 10 m walking tests are warranted. Secondly, in the second physical examination, some HCWs only tested the seropositivity of antibodies and did not perform the titre test. Therefore, only the antibody titres between the first and third physical examinations were compared. More studies should be carried out in the follow-up to further explore the dynamic changes of antibody levels from the acute stage to longer period after discharge. Thirdly, in analysing the dynamic changes of lymphocyte subsets, only the relative numbers of lymphocyte subsets instead of absolute numbers were investigated. In future studies, the dynamic changes of the absolute numbers of lymphocyte subsets over time after discharge should also be explored. Fourthly, 333 health care workers with severe COVID-19 were included in the cohort; however, different sample sizes are included for each variable and at each time point assessment. The results of this study need to be verified in larger sample size researches though this study has included the HCWs in Hubei Province, which has the largest proportion of HCWs with severe COVID-19 in China, and no significant differences were found in characteristics of the participants included and excluded in this study. Fifthly, given the limited sample sizes, stratified analyses according to gender and body mass index were not performed further in this study. In the future, studies focusing on the health consequences after discharge in HCWs with severe COVID-19 of different genders and body mass indexes are warranted.

## Conclusions

The HCWs with severe COVID-19 showed improvement in functional fitness within 1 year after discharge, active intervention should be applied to help their recovery if necessary. It is of vital significance to continue monitoring the functional fitness, antibodies to SARS-CoV-2 and immunological indicators after 1 year of discharge from hospital in HCWs with severe COVID-19.

## Supplementary Information

**Additional file 1: Table S1**. Demographic characteristics of HCWs included and excluded in this study. **Table S2**. Distribution of cytokines in three physical examinations. **Table S3**. Distribution of cytokines before discharge and in three physical examinations. **Table S4**. Levels of cytokines before discharge and in three physical examinations. **Table S5**. Distribution of lymphocyte subsets in three physical examinations. **Table S6**. Distribution of lymphocyte subsets before discharge and in three physical examinations. **Table S7**. Relative numbers of lymphocyte subsets before discharge and in three physical examinations. **Table S8**. Pearson correlations between indicators of cytokines and lymphocyte subsets (N=508). **Figure S1**. Number of HCWs with COVID-19 discharged from hospitals across time. **Figure S2**. Antibody titres against SARS-CoV-2 among 136 HCWs who had titres of antibodies both in first and third physical examinations. **Figure S3**. Levels of cytokines categorized by the time of physical examination in 114 HCWs who competed all three physical examinations. **Figure S4**. Relative numbers of lymphocyte subsets categorized by the time of physical examination in 114 HCWs who competed all three physical examinations

## Data Availability

All data generated or analysed during this study are included in this published article [and its supplementary information files]. Since the cohort is still going on, we may not make the data available to others.

## Abbreviations

BMI: Body mass index
COVID-19: Novel coronavirus pneumonia
HCWs: Health care workers
ICU: Intensive care unit admission
SARS-CoV-2: Severe acute respiratory syndrome coronavirus 2
