# Supplementary material for: Dynamic changes of functional fitness, antibodies to SARS-CoV-2 and immunological indicators within 1 year after discharge in Chinese health care workers with severe COVID-19: a cohort study
Source: BMC Med. 2021 Jul 14;19:163. doi: 10.1186/s12916-021-02042-0 (PMC8277525; doi:10.1186/s12916-021-02042-0)
Supplement: Supplementary file 1 — Additional file 1: Table S1. Demographic characteristics of HCWs included and excluded in this study. Table S2. Distribution of cytokines in three physical examinations. Table S3. Distribution of cytokines before discharge and in three physical examinations. Table S4. Levels of cytokines before discharge and in three physical examinations. Table S5. Distribution of lymphocyte subsets in three physical examinations. Table S6. Distribution of lymphocyte subsets before discharge and in three physical examinations. Table S7. Relative numbers of lymphocyte subsets before discharge and in three physical examinations. Table S8. Pearson correlations between indicators of cytokines and lymphocyte subsets (N=508). Figure S1. Number of HCWs with COVID-19 discharged from hospitals across time. Figure S2. Antibody titres against SARS-CoV-2 among 136 HCWs who had titres of antibodies both in first and third physical examinations. Figure S3. Levels of cytokines categorized by the time of physical examination in 114 HCWs who competed all three physical examinations. Figure S4. Relative numbers of lymphocyte subsets categorized by the time of physical examination in 114 HCWs who competed all three physical examinations [file 12916_2021_2042_MOESM1_ESM.doc]

**Additional file 1**

**Title: Dynamic changes of functional fitness, antibodies to SARS-CoV-2 and immunological indicators within one year after discharge in Chinese health care workers with severe COVID-19: a cohort study**

**Table S1-Demographic characteristics of HCWs included and excluded in this study**

| **Characteristics** | **All (N=656)** | **Included (N=333)** | **Excluded (N=323)** |
| --- | --- | --- | --- |
| **Demographic characteristics** | |  |  |
| Age (years) | 35.0 (30.0-42.0) | 36.0 (31.0- 43.0) | 34.0 (28.3-40.0) |
| Sex |  |  |  |
| Male | 151 (23%) | 76 (23%) | 75 (23%) |
| Female | 505 (77%) | 257 (77%) | 248 (77%) |
| Education |  |  |  |
| High school and lower | 131/618 (21%) | 75/327 (23%) | 56/291 (19%) |
| College and higher | 487/618 (79%) | 252/327 (77%) | 235/291 (81%) |
| Roles in work |  |  |  |
| Doctors | 155 (24%) | 89 (27%) | 66 (20%) |
| Nurses | 378 (58%) | 191 (57%) | 187 (58%) |
| Other | 123 (19%) | 53 (16%) | 70 (22%) |
| BMI (kg/m^2^) | 22.7 (20.5-25.2) | 23.5 (21.1-25.9) | 21.9 (19.9-24.3) |
| Smoke habit |  |  |  |
| No | 616/634 (97%) | 326 (98%) | 290/301 (96%) |
| Yes | 18/634 (3%) | 7 (2%) | 11/301 (4%) |

Data are n (%), n/N (%), or median (IQR). The differing denominators used indicate missing data. BMI=body mass index.

**Table S2-Distribution of cytokines in three physical examinations**

| **Categories** | **IFN-γ (pg/mL)** | **IL-10 (pg/mL)** | **IL-2**  **(pg/mL)** | **IL-4**  **(pg/mL)** | **IL-6**  **(pg/mL)** | **TNF-α**  **(pg/mL)** |
| --- | --- | --- | --- | --- | --- | --- |
| First physical examination (N=183) | | | | | | |
| Elevated* | 1 (0.5%) | 10 (5.5%) | 35 (19.1%) | 143 (78.1%) | 130 (71.0%) | 93 (50.8%) |
| Normal range† | 0.98-9.13 | 2.80-5.00 | 1.60-4.10 | 2.19-3.20 | 3.44-5.23 | 2.57-21.95 |
| Decreased* | 0 (0.0%) | 0 (0.0%) | 0 (0.0%) | 0 (0.0%) | 0 (0.0%) | 0 (0.0%) |
| Second physical examination (N=166) | | | | | | |
| Elevated* | 1 (0.6%) | 3 (1.8%) | 1 (0.6%) | 0 (0.0%) | 110 (66.3%) | 124 (74.7%) |
| Normal range† | 2.04-8.75 | 2.16-4.68 | 1.30-3.43 | 1.98-2.91 | 3.27-5.27 | 2.10-4.52 |
| Decreased* | 0 (0.0%) | 0 (0.0%) | 0 (0.0%) | 0 (0.0%) | 0 (0.0%) | 0 (0.0%) |
| Third physical examination (N=159) | | | | | | |
| Elevated* | 2 (1.3%) | 1 (0.6%) | 4 (2.5%) | 2 (1.3%) | 67 (42.1%) | 73 (45.9%) |
| Normal range† | 0.98-9.13 | 1.04-3.11 | 0.13-1.75 | 0.55-2.20 | 0.52-5.20 | 0.98-4.49 |
| Decreased* | 0 (0.0%) | 0 (0.0%) | 0 (0.0%) | 0 (0.0%) | 0 (0.0%) | 0 (0.0%) |

*Data are n (%). † Data are shown as the normal ranges of the indicators.

**Table S3-Distribution of cytokines before discharge and in three physical examinations**

| **Categories** | **IFN-γ (pg/mL)** | **IL-10 (pg/mL)** | **IL-2**  **(pg/mL)** | **IL-4**  **(pg/mL)** | **IL-6**  **(pg/mL)** | **TNF-α**  **(pg/mL)** |
| --- | --- | --- | --- | --- | --- | --- |
| Before discharge (N=30) | | | | | | |
| Elevated* | 0 (0.0%) | 6 (20.0%) | 0 (0.0%) | 0 (0.0%) | 16 (53.3%) | 0 (0.0%) |
| Normal range† | 1.12-4.69 | 2.22-4.99 | 2.18-3.73 | 1.24-2.93 | 2.33-5.25 | 1.46-15.70 |
| Decreased* | 0 (0.0%) | 0 (0.0%) | 0 (0.0%) | 0 (0.0%) | 0 (0.0%) | 0 (0.0%) |
| First physical examination (N=26) |  |  |  |  |  |  |
| Elevated* | 0 (0.0%) | 1 (3.8%) | 4 (15.4%) | 21 (80.8%) | 18 (69.5%) | 14 (53.8%) |
| Normal range† | 2.62-4.69 | 2.95-5.00 | 3.00-4.10 | 2.65-3.19 | 3.44-5.04 | 2.76-19.09 |
| Decreased* | 0 (0.0%) | 0 (0.0%) | 0 (0.0%) | 0 (0.0%) | 0 (0.0%) | 0 (0.0%) |
| Second physical examination (N=20) | | | | | | |
| Elevated* | 0 (0.0%) | 0 (0.0%) | 0 (0.0%) | 0 (0.0%) | 11 (55.0%) | 14 (70.0%) |
| Normal range† | 2.08-2.93 | 2.26-4.03 | 1.33-2.45 | 2.04-2.87 | 3.33-5.19 | 2.16-3.62 |
| Decreased* | 0 (0.0%) | 0 (0.0%) | 0 (0.0%) | 0 (0.0%) | 0 (0.0%) | 0 (0.0%) |
| Third physical examination (N=22) | | | | | | |
| Elevated* | 0 (0.0%) | 0 (0.0%) | 0 (0.0%) | 0 (0.0%) | 8 (36.4%) | 8 (36.4%) |
| Normal range† | 1.12-1.63 | 1.44-2.26 | 0.13-1.34 | 1.16-1.79 | 1.45-4.62 | 0.98-3.25 |
| Decreased* | 0 (0.0%) | 0 (0.0%) | 0 (0.0%) | 0 (0.0%) | 0 (0.0%) | 0 (0.0%) |

Data are n (%). †Data are shown as the normal ranges of the indicators.

**Table S4-Levels of cytokines before discharge and in three physical examinations**

| **Categories** | **IFN-γ (pg/mL)** | **IL-10 (pg/mL)** | **IL-2**  **(pg/mL)** | **IL-4**  **(pg/mL)** | **IL-6**  **(pg/mL)** | **TNF-α**  **(pg/mL)** |
| --- | --- | --- | --- | --- | --- | --- |
| Before discharge (N=30) | 2.24 (1.91-2.73) | 4.23 (3.29-4.94) | 2.62 (2.43-2.72) | 1.95 (1.59-2.25) | 5.89 (3.50-13.87) | 2.09 (1.80-2.31) |
| First physical examination (N=26) | 3.98 (3.80-4.34)* | 4.01 (3.89-4.44) | 3.94 (3.84-4.08)* | 3.53 (3.30-3.72)* | 5.87 (4.94-9.33) | 23.08 (4.24-37.28)* |
| Second physical examination (N=20) | 2.54 (2.36- 2.75) | 3.40 (2.91-3.54)† | 1.93 (1.53-2.18)† | 2.47 (2.30-2.55)* | 5.47 (4.64-9.81) | 8.45 (3.32-15.10)* |
| Third physical examination (N=22) | 1.35 (1.26-1.45)† | 1.69 (1.54-1.82)† | 0.84 (0.75-1.08)† | 1.63 (1.55-1.73)† | 3.01 (2.32-9.20)† | 2.76 (2.11-6.89)* |

Data are median (IQR). The median levels of cytokines before discharge and in follow-up physical examination was compared using Mann–Whitney U test. *The cytokine levels at that time point were statistically higher than levels before discharge, *P* <0.05. †The cytokine levels at that time point were statistically lower than levels before discharge, *P* <0.05.

**Table S5-Distribution of lymphocyte subsets in three physical examinations**

| **Categories** | **B cells**  **(%)** | **CD3^+^ T cells (%)** | **CD4^+^ T cells (%)** | **CD4^+^/CD8^+^ cell ratio** | **CD8^+^ T cells (%)** | **NK cells**  **(%)** |
| --- | --- | --- | --- | --- | --- | --- |
| First physical examination (N=183) | | | | | | |
| Elevated* | 2 (1.1%) | 4 (2.2%) | 3 (1.6%) | 4 (2.2%) | 18 (9.8%) | 17 (9.3%) |
| Normal range† | 4.37-18.12 | 58.21-83.71 | 25.80-50.24 | 0.45-2.48 | 14.31-38.79 | 3.97-30.13 |
| Decreased* | 9 (4.9%) | 16 (8.7%) | 8 (4.4%) | 1 (0.5%) | 1 (0.5%) | 0 (0.0%) |
| Second physical examination (N=166) | | | | | | |
| Elevated* | 5 (3.0%) | 3 (1.8%) | 3 (1.8%) | 4 (2.4%) | 14 (8.4%) | 13 (7.8%) |
| Normal range† | 4.13-18.07 | 58.83-82.64 | 25.42-50.15 | 0.41-2.62 | 14.54-38.52 | 3.55-30.31 |
| Decreased* | 3 (1.8%) | 15 (9.0%) | 9 (5.4%) | 0 (0.0%) | 1 (0.6%) | 0 (0.0%) |
| Third physical examination (N=159) | | | | | | |
| Elevated* | 4 (2.5%) | 2 (1.3%) | 1 (0.6%) | 4 (2.5%) | 9 (5.7%) | 18 (11.3%) |
| Normal range† | 4.15-17.61 | 58.51-84.10 | 25.91-51.10 | 0.42-2.69 | 15.16-38.92 | 3.79-29.34 |
| Decreased* | 3 (1.9%) | 19 (11.9%) | 10 (6.3%) | 0 (0.0%) | 3 (1.9%) | 0 (0.0%) |

Data are n (%). †Data are shown as the normal ranges of the indicators.

**Table S6-Distribution of lymphocyte subsets before discharge and in three physical examinations**

| **Categories** | **B cells**  **(%)** | **CD3^+^ T cells (%)** | **CD4^+^ T cells (%)** | **CD4^+^/CD8^+^ cell ratio** | **CD8^+^ T cells (%)** | **NK cells**  **(%)** |
| --- | --- | --- | --- | --- | --- | --- |
| Before discharge (N=28) | | | | | | |
| Elevated* | 2 (7.1%) | 3 (10.7%) | 1 (3.6%) | 1 (17.9%) | 3 (10.7%) | 0 (0.0%) |
| Normal range† | 4.51-16.53 | 63.80-82.66 | 28.3-51.36 | 0.69-2.63 | 19.80-37.95 | 3.57-30.42 |
| Decreased* | 0 (0.0%) | 3 (10.7%) | 2 (7.1%) | 0 (0.0%) | 0 (0.0%) | 5 (17.9%) |
| First physical examination (N=26) |  |  |  |  |  |  |
| Elevated* | 1 (3.8%) | 1 (3.8%) | 0 (0.0%) | 0 (0.0%) | 3 (11.5%) | 4 (15.4%) |
| Normal range† | 4.37-15.69 | 59.73-82.97 | 26.87-45.37 | 0.58-2.43 | 14.45-37.29 | 7.16-25.87 |
| Decreased* | 0 (0.0%) | 3 (11.5%) | 1 (3.8%) | 0 (0.0%) | 0 (0.0%) | 0 (0.0%) |
| Second physical examination (N=20) | | | | | | |
| Elevated* | 1 (5.0%) | 0 (0.0%) | 1 (5.0%) | 0 (0.0%) | 1 (5.0%) | 4 (20.0%) |
| Normal range† | 4.86-18.07 | 58.83-82.64 | 26.20-45.69 | 0.61-2.18 | 20.41-36.77 | 8.27-27.05 |
| Decreased* | 0 (0.0%) | 2 (10.0%) | 2 (5.0%) | 0 (0.0%) | 1 (5.0%) | 0 (0.0%) |
| Third physical examination (N=22) | | | | | | |
| Elevated* | 2 (9.1%) | 0 (0.0%) | 0 (0.0%) | 0 (0.0%) | 1 (4.5%) | 3 (13.6%) |
| Normal range† | 4.16-14.50 | 58.90-84.10 | 25.91-44.77 | 0.71-2.16 | 18.74-36.90 | 7.08-27.35 |
| Decreased* | 1 (4.5%) | 4 (18.2%) | 2 (9.1%) | 0 (0.0%) | 0 (0.0%) | 0 (0.0%) |

*Data are n (%). †Data are shown as the normal ranges of the indicators.

**Table S7-Relative numbers of lymphocyte subsets before discharge and in three physical examinations**

| **Categories** | **B cells**  **(%)** | **CD3^+^ T cells (%)** | **CD4^+^ T cells (%)** | **CD4^+^/CD8^+^ cell ratio** | **CD8^+^ T cells (%)** | **NK cells**  **(%)** |
| --- | --- | --- | --- | --- | --- | --- |
| Before discharge (N=28) | 12.41 (7.75-15.33) | 75.57 (70.69-80.17) | 38.72 (35.13-43.59) | 1.35 (1.07-1.80) | 27.8 (24.01-34.66) | 7.74 (4.02-11.49) |
| First physical examination (N=26) | 10.21 (7.96-11.92) | 69.11 (62.61-76.77) | 33.62 (31.16-38.30)† | 1.33 (1.03-1.53) | 25.29 (22.53-34.43) | 17.29 (12.08-23.58)* |
| Second physical examination (N=20) | 10.07 (7.43-13.30) | 67.45 (60.80-72.50) | 33.10 (30.69-36.90)† | 1.31 (1.08-1.57) | 26.46 (21.42-29.94) | 18.58 (13.78-25.52)* |
| Third physical examination (N=22) | 10.13 (8.50-12.92) | 67.63 (61.76-72.27) | 31.54 (29.66-35.54)† | 1.34 (1.01-1.48) | 26.25 (21.58-30.31) | 19.37 (12.74-23.47)* |

Data are median (IQR). The median values of relative numbers of B cells, CD4^+^ T cells, CD8^+^ T cells and NK cells before discharge and in follow-up physical examination were compared using Mann–Whitney U test. The median values of relative numbers of of CD3^+^ T cells and CD4^+^/CD8^+^ cell ratio before discharge and in follow-up physical examination were compared using t test. *The relative numbers of lymphocyte subsets at that time point were statistically higher than that before discharge, *P* <0.05. †The relative numbers of lymphocyte subsets at that time point were statistically lower than that before discharge, *P* <0.05.

**Table S8-Pearson correlations between indicators of cytokines and lymphocyte subsets (N=508)**

| **Categories** | **B cells**  **(%)** | **CD3^+^ T cells (%)** | **CD4^+^ T cells (%)** | **CD4^+^/CD8^+^ cell ratio** | **CD8^+^ T cells (%)** | **NK cells**  **(%)** |
| --- | --- | --- | --- | --- | --- | --- |
| **IFN-γ (pg/mL)** | -0.052 | -0.050 | -0.090 | -0.080 | 0.026 | 0.097 |
| **IL-10 (pg/mL)** | 0.067 | 0.027 | 0.065 | 0.097 | -0.051 | -0.057 |
| **IL-2 (pg/mL)** | -0.086 | -0.008 | -0.063 | -0.073 | 0.044 | 0.083 |
| **IL-4 (pg/mL)** | -0.062 | 0.079 | 0.024 | -0.046 | 0.078 | -0.007 |
| **IL-6 (pg/mL)** | -0.071 | 0.011 | -0.044 | -0.036 | 0.036 | 0.021 |
| **TNF-α (pg/mL)** | -0.054 | 0.013 | 0.013 | 0.020 | 0.002 | 0.020 |

Data are r values calculated by pearson correlations between levels of cytokines and levels of lymphocyte subsets.





**Figure S1-Number of HCWs with COVID-19 discharged from hospitals across time**

HCWs=health care workers.





**Figure S2-Antibody titres against SARS-CoV-2 among 136 HCWs who had titres of antibodies both in first and third physical examinations**

A:The titres of IgM in the first and third physical examination. B:The titres of IgG in the first and third physical examination. Data are shown as min, Q25, Q50, Q75 and max value. The comparison of titres of IgM and IgG in the first and third physical examination was performed with Mann–Whitney U test. The sample sizes in the first and third physical examination were both 136. **P* <0.001.





**Figure S3-Levels of cytokines categorized by the time of physical examination in 114 HCWs who competed all three physical examinations**

A:IFN-γ, B:IL-10, C:IL-2, D:IL-4, E:IL-6, F:TNF-α. The median levels of cytokines in the first, second and third physical examination were compared using Kruskal–Wallis test. The sample sizes in the first, second and third physical examination were all 114. Data are shown as min, Q25, Q50, Q75 and max value. **P* <0.001.

**

**

**Figure S4-Relative numbers of lymphocyte subsets categorized by the time of physical examination in 114 HCWs who competed all three physical examinations**

A:B cells, B:CD3^+^ T cells, C:CD4^+^ T cells, D:CD4^+^/CD8^+^ cell ratio, E:CD8^+^ T cells, F:NK cells. The median relative numbers of lymphocyte subsets in the first, second and third physical examination were compared using Kruskal–Wallis test. The sample sizes in the first, second and third physical examination were all 114. Data are shown as min, Q25, Q50, Q75 and max value.
